# Public health-relevant consequences of the COVID-19 pandemic on malaria in sub-Saharan Africa: a scoping review

**DOI:** 10.1186/s12936-021-03872-2

**Published:** 2021-08-11

**Authors:** Anna-Katharina Heuschen, Guangyu Lu, Oliver Razum, Alhassan Abdul-Mumin, Osman Sankoh, Lorenz von Seidlein, Umberto D’Alessandro, Olaf Müller

**Affiliations:** 1 Institute of Global Health, Medical School, Ruprecht-Karls-University, Heidelberg, Germany; 2 Department of Public Health, Medical College, Yangzhou University, Yangzhou, China; 3 Department of Epidemiology and International Public Health, School of Public Health, Bielefeld University, Bielefeld, Germany; 4 Department of Paediatrics and Child Health, School of Medicine, University for Development Studies, Tamale, Ghana; 5 Statistics Sierra Leone, Tower Hill, Freetown, Sierra Leone; 6 School of Public Health, Faculty of Health Sciences, University of the Witwatersrand, Johannesburg, South Africa; 7 Mahidol Oxford Tropical Medicine Research Unit, Faculty of Tropical Medicine, Mahidol University, Bangkok, Thailand; 8 MRC The Gambia, Serekunda, The Gambia

**Keywords:** COVID-19, Coronavirus, Malaria, Pandemic, Sub-Saharan Africa, Public health, Global health

## Abstract

**Background:**

The COVID-19 pandemic has resulted in unprecedented challenges to health systems worldwide, including the control of non-COVID-19 diseases. Malaria cases and deaths may increase due to the direct and indirect effects of the pandemic in malaria-endemic countries, particularly in sub-Saharan Africa (SSA). This scoping review aims to summarize information on public health-relevant effects of the COVID-19 pandemic on the malaria situation in SSA.

**Methods:**

Review of publications and manuscripts on preprint servers, in peer-reviewed journals and in grey literature documents from 1 December, 2019 to 9 June, 2021. A structured search was conducted on different databases using predefined eligibility criteria for the selection of articles.

**Results:**

A total of 51 papers have been included in the analysis. Modelling papers have predicted a significant increase in malaria cases and malaria deaths in SSA due to the effects of the COVID-19 pandemic. Many papers provided potential explanations for expected COVID-19 effects on the malaria burden; these ranged from relevant diagnostical and clinical aspects to reduced access to health care services, impaired availability of curative and preventive commodities and medications, and effects on malaria prevention campaigns. Compared to previous years, fewer country reports provided data on the actual number of malaria cases and deaths in 2020, with mixed results. While highly endemic countries reported evidence of decreased malaria cases in health facilities, low endemic countries reported overall higher numbers of malaria cases and deaths in 2020.

**Conclusions:**

The findings from this review provide evidence for a significant but diverse impact of the COVID-19 pandemic on malaria in SSA. There is the need to further investigate the public health consequences of the COVID-19 pandemic on the malaria burden.

Protocol registered on Open Science Framework: https://doi.org/10.17605/OSF.IO/STQ9D

## Background

The emergence of SARS-CoV-2 in China by the end of 2019 has led to the largest pandemic in recent human history [1, 2]. By 14 June, 2021, there were some 176 million confirmed cases of COVID-19, including 3.8 million deaths, reported to the World Health Organization (WHO) [3]. The COVID-19 epidemic waves show variable dynamics in the different WHO Regions, with the highest burden in the American, European and Southeast Asian Regions [3, 4]. The latter has recently shown particularly high incidence rates, and India is now reporting the second highest number of confirmed cases after the USA [3]. In contrast, the African and the Western Pacific WHO Regions continue to report only relatively low numbers of cases and deaths [3, 4].

It was initially predicted that Africa would be the worst affected region by the COVID-19 pandemic because of its weak health systems, prevailing poverty and the high burden of other infectious diseases [5, 6]. However, by the end of 2020 about 3.5% of the global number of COVID-19 cases and deaths were reported from this continent, which is home to 17% of the world’s population [3, 7]. Overall, the epidemiology of COVID-19 in Africa remains puzzling [5]. By 14 June, 2021, there were some 3.6 million COVID-19 cases and 89,000 deaths reported from the entire continent, and most of these were from its northern and southern regions [8, 9]. Potential explanations for such a situation are incomplete data due to much lower testing capacities, a significantly younger population, overall lower population mobility, cross-reactive immunity or immunomodulation due to high prevalence of other infectious agents, and effects of public health responses [5, 7, 10]. First findings from SARS-CoV-2 seroprevalence surveys support the evidence for significant under-reporting and for a predominance of asymptomatic and mild cases [11, 12]. Nevertheless, it appears that the second epidemic wave has hit the African continent more severely than the first one, possibly explained by the emergence of more transmissible SARS-CoV-2 variants [7, 13].

Globally, malaria is still the most important parasitic disease and responsible for a quarter of all deaths among children under 5 years old in sub-Saharan Africa (SSA) [14, 15]. The efforts for global malaria control and elimination have achieved large successes during the last two decades, but progress has stalled in recent years, and the COVID-19 pandemic could largely reverse the overall trend [16, 17]. This review aims to summarize currently available data and understanding of the direct and indirect effects of the COVID-19 pandemic on the malaria burden in SSA.

## Methods

### Search strategy and selection criteria

Due to the complex topic and the different type of studies available, a scoping review methodology was chosen [18]. The study protocol (published on OFS, https://doi.org/10.17605/OSF.IO/STQ9D) complies with the ‘Preferred Reporting Items for Systematic Reviews and Meta-Analyses for Scoping Reviews (PRISMA-ScR) checklist’ [19]. The following inclusion criteria were applied: papers needed to respect the categories of the PICo-framework (Problem: malaria situation; Interest: the public health impact of the COVID-19 pandemic; Context: sub-Saharan Africa) [20]. No restrictions regarding the study type and the publication status were applied. Possible languages were English, French and German; papers published between 1 December, 2019 and 9 June, 2021 were included. In line with the protocol, the search strategy was developed, and the following databases were searched: PubMed; Ovid MEDLINE(R); Web of Science; Biosis Previews; MedRxiv, and *The Lancet*. Grey literature was included using WHO database and Google Scholar. Three broad blocks of search terms were used: (1) COVID-19; (2) malaria; (3) sub-Saharan Africa. The detailed search strategy is available in Appendix 1.

For the extracted findings, two researchers (OM and AH) conducted independently the title screening, then the abstract screening and finally the full text review. The papers selected for full-text reading were assessed for eligibility; ineligible papers did not include information on public health-relevant consequences of the COVID-19 pandemic on malaria in SSA. Inclusion decisions depended on whether the paper agreed to the PICo-framework and the formal eligibility criteria. Results were compared after each step for discussion and for reaching a consensus. For the analysis of the finally included papers, a data extraction table was constructed (Appendix 2).

The following information was extracted from the papers: authors, title, study place, study population, study design, and outcome. Moreover, the papers were categorized by study type: modelling study, report (country report, general report, case report), review, opinion paper, and policy guideline. The information content was structured and analysed around the following themes:

- Modelled impact of COVID-19 on malaria.
- Diagnostical and clinical aspects.
- Access to health care services.
- Availability of curative and preventive malaria commodities.
- Impact on malaria programmes.
- Epidemiologic data from countries.

**Fig. 1:**
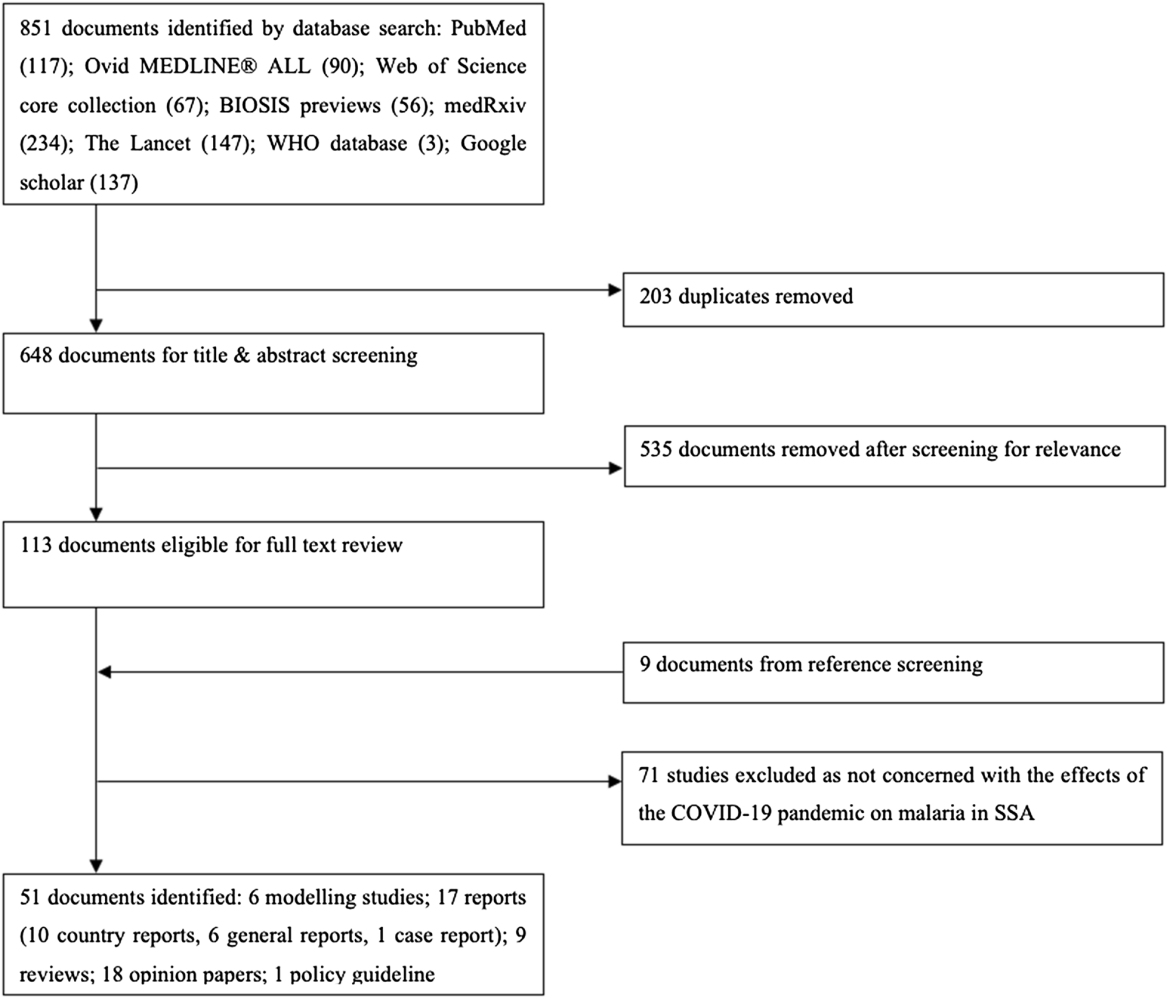
Conceptual framework presenting the different factors resulting from the global COVID-19 pandemic on the malaria situation in sub-Saharan Africa

Based on these findings, a conceptual framework was created, with input from all co-authors (Fig. 1).

## Results

**Fig. 2:**
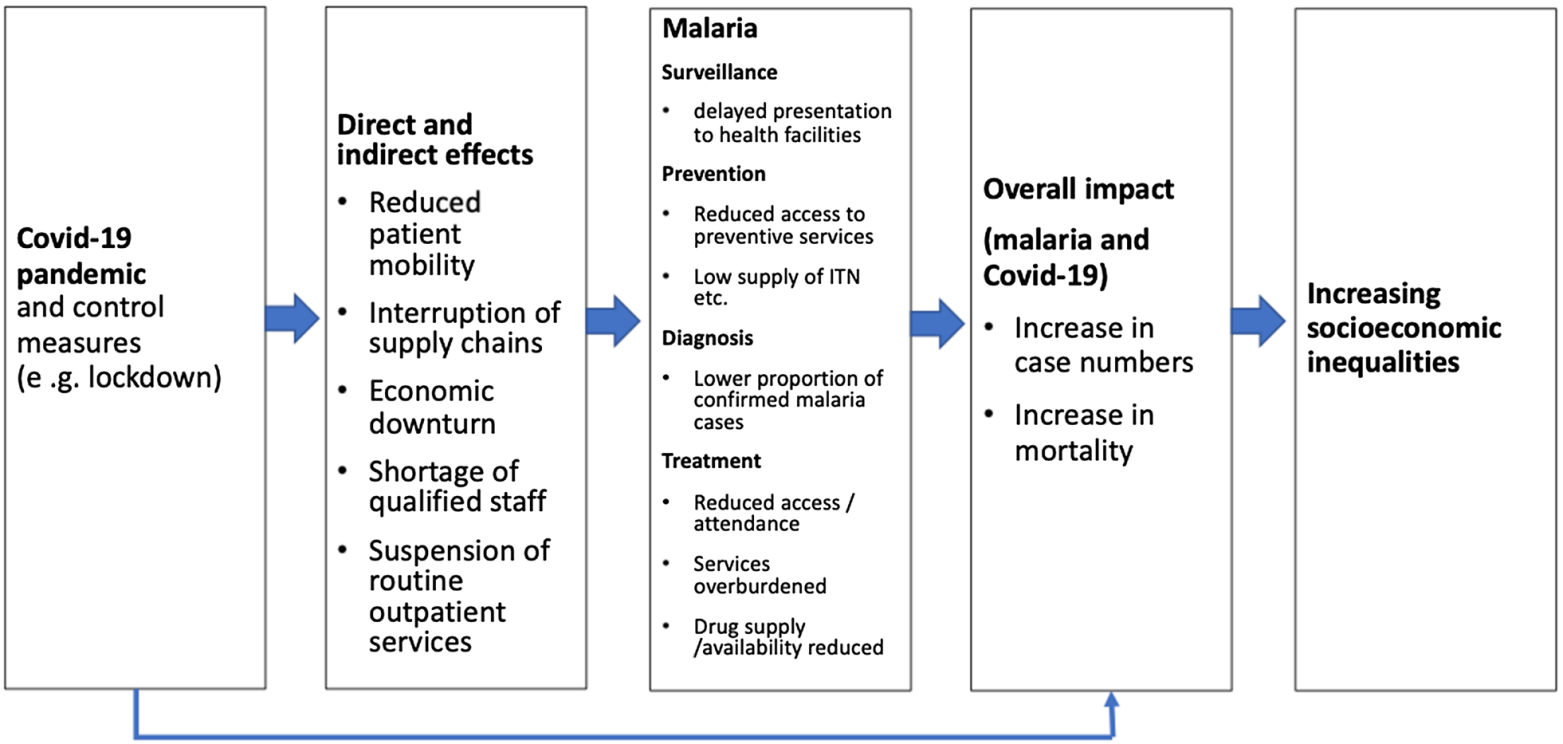
Study selection process

Figure 2 visualizes the study selection process. The initial search produced 851 documents. After removal of 203 duplicates, 648 documents underwent title and abstract screening. After exclusion of 535 documents, which did not meet the inclusion criteria, 113 papers were included for full text review. Nine papers were added from reference screening; 71 were excluded as they also did not meet the inclusion criteria. Thus, a total of 51 papers were reviewed (6 modelling studies, 10 country reports, 6 general reports, 1 case report, 9 review papers, 18 opinion papers, and 1 policy guideline).

### Modelled impact of COVID-19 on malaria

Five papers predicted the evolution of the malaria burden in SSA based on different potential scenarios. Considering primarily a reduced access to effective anti-malarial treatment and reduced insecticide-treated mosquito net (ITN) distribution, Weiss et al. predicted in their worst-case scenario (75% fewer anti-malarial drugs and ITNs) and for the year 2020 that in SSA countries, malaria cases would increase by 22% (from 215 to 262 million) and malaria deaths by 99% (from 386,000 to 769,000); the lower access to anti-malarial treatment had a larger effect than reduced ITN distribution [21]. These estimates mirror those by the WHO, but the authors described the effects of nine different scenarios compared to the effects of three scenarios by Weiss et al. [22]. Comparable estimates were published by Sherrard-Smith et al. for the scenario of complete interruption of ITN distribution and 50% decreased access to anti-malarials, they predicted malaria deaths would increase in SSA to 779,000 for the year 2020 [23]. A further analysis by WHO predicted up to 100,000 additional deaths in 2020 with a 50% lower access to anti-malarials [17]. However, all these authors emphasized that the projected effects on malaria services and mortality are highly uncertain because these estimates are heavily dependent on how countries respond to the COVID-19 pandemic. Regarding the relative burden of COVID-19 in Africa, one study concluded that the excess disability-adjusted life years (DALYs) lost by malaria due to COVID-19 may exceed those directly lost due to COVID-19 [24].

### Diagnostical and clinical aspects

The clinical manifestations of COVID-19 and malaria largely overlap; fever, headache, joint pain, respiratory symptoms, and general weakness are frequently seen with both diseases [25–27]. Thus, diagnosis based on symptoms alone can result in inadequate treatment, with potentially harmful consequences. Untreated malaria can be rapidly fatal and COVID-19 patients must be quarantined to interrupt community transmission [14, 28]. Despite increasing availability of rapid diagnostic tests (RDTs) for malaria in all endemic areas, presumptive diagnosis of malaria is still common in SSA, and the WHO Malaria Technical Guidelines adapted to COVID-19 confirm this situation [29, 30]. Initial information available for 2020 suggests major disruptions in malaria diagnosis and treatment due to COVID-19 [31, 32].

Human travel history is important for SARS-CoV-2 and malaria, as for both of them asymptomatic persons can spread and/or maintain transmission of the infectious agent [26]. Malaria might have been reduced by the COVID-19 movement restrictions, especially in heterogenous malaria-endemic settings where transmission frequently results from migration flows of infected individuals across different regions [33]. Moreover, malaria and SARS-CoV-2 co-infections may be associated with clinical disease modification, although data on this are limited [27, 34–36].

While symptomatic malaria affects mainly children and younger age groups in endemic areas, COVID-19 affects all age groups but is more frequently symptomatic and severe with increasing age [34]. However, in areas of low malaria transmission, the age groups affected by the two diseases largely overlap [37]. RDTs are essential for malaria diagnosis in rural SSA and may also become important for COVID-19, as the PCR test capacity is very limited [32]. However, the impact of rather low sensitivity and specificity of COVID-19 RDTs is still under intense discussion [27, 38]. An additional challenge for differential diagnosis is the increasing frequency of gene-mutated *Plasmodium* parasites, especially in the Horn of Africa, that escape detection by standard RDTs [37].

The role of anti-malarials, e.g. artemisinin derivates and chloroquine (CQ), in the COVID-19 pandemic is complex. Various artemisinin derivates, artemisinin-based combination therapy (ACT) as well as CQ have been shown to be effective against SARS-CoV-1 and SARS-CoV-2 in vitro [39–42]. However, such beneficial effect was not confirmed by several clinical trials [43–46]. The wide use of these treatments in highly malaria-endemic countries has been suggested to be responsible for the reported low COVID-19 burden in SSA [36, 47]. On the other hand, the increased usage of these drugs for COVID-19 prevention and treatment in some malaria-endemic countries might have reduced malaria [25]. A frequent off-label use of artemisinin-based drugs may also increase the likelihood of emerging drug resistance and thus threatens the most important of the remaining effective anti-malarials [33, 48–50].

### Access to health care services

The COVID-19 pandemic in SSA endangers access to health care services due to several factors. Direct factors include restricted services and closures of health facilities because of reduced health care worker (HCW) capacity due to lack of personal protective equipment (PPE), stigmatization, fear of getting infected, or absence due to COVID-19 quarantine, disease or death [27, 32, 50, 51]. Delayed treatment results in prolonged gametocyte carriage and additional opportunities for transmission.

Moreover, because of overload of COVID-19 patients and consequently reduced time to manage other diseases, or due to movement and travel restrictions and for fear of becoming infected with COVID-19, sick individuals with diseases other than COVID-19 no longer attend health facilities [33, 48, 52]. As older people fear severe COVID-19 disease and may thus avoid visiting health facilities, this might affect children the most as they depend on their care givers if sick, including for malaria [35, 49]. Stay-at-home advice for febrile diseases, especially at the beginning of the pandemic, enhanced such behaviour [17, 33].

Indirect factors include reduced income during lockdowns due to inability to perform informal work, and subsequently reduced purchasing power [52]. The resulting increase in poverty leads to challenges for paying the costs for routine care, drugs or transportation fees [51]. Lockdowns, movement restrictions and border closures further complicate access to health facilities and have also threatened the functioning of malaria surveillance systems [16, 28, 33, 51]. Institutional mistrust and lack of valid information further reduced visits to health care facilities and reduced uptake of preventive measures; as an example, myths about the spread of COVID-19 via ITNs led to a reduced usage of this essential intervention in Sierra Leone [25].

### Availability of curative and preventive commodities and medicines

Increased material costs, reluctance of producers to invest, travel restrictions, border closures, and lockdowns resulted in a lower availability of medical malaria products [26, 28, 48]. Low- and middle-income countries (LMICs) are disproportionately affected as they essentially rely on importation of these commodities [52]. Excessive use of anti-malarials for COVID-19 prevention and treatment in some regions has led to shortages for their original purpose [17, 30]. Some international companies switched from the production of malaria products to COVID-19 products [48, 49, 51]. Difficult access to health facilities lowered the availability of essential drugs and increased their price, with subsequent increases in purchase and usage of sub-standard drugs and alternative medicines [28, 51–53]. In addition, PPE needed for the implementation of different malaria services [e.g., indoor residual spraying of insecticides (IRS)] has become scarce and expensive on global markets [17, 54].

### Impact of the pandemic on malaria programmes

The extent of the pandemic’s impact on malaria depends on the timing of its waves. The largest effects may occur if the COVID-19 transmission peaks and the planned malaria campaigns overlap [21, 23, 53, 55]. About three-quarters of malaria-affected countries reported disruptions of malaria services and programmes [17, 32, 33, 50, 53, 56–58]. Re-allocation of funds from other disease control programmes to the control of COVID-19 have been common and pose great problems for malaria control [30, 32, 35, 59, 60]. Ongoing malaria programmes (e.g., IRS, ITN interventions) need to be adapted to the restrictions associated with COVID-19 control measures, which requires additional financial resources [32, 33, 36]. Programmes for vulnerable populations living in remote areas are particularly at risk as they strongly depend on logistics and external financing [33, 48]. Disrupted ITN programmes will lead to increased malaria transmission as 80% of the nets are distributed through mass campaigns [22, 48, 53, 55]. IRS campaigns face many challenges as they require direct household contact [33, 50, 57].

Nevertheless, these challenges have led to new approaches: Benin digitalized its ITN mass distribution campaign using a ‘no touch’ payment for campaign workers. The national strategy was changed from a fixed-point to a door-to-door-distribution procedure, which enabled health workers to provide additional community health education on COVID-19 and other aspects; other countries followed the Benin model and by the end of 2020, 90% of all globally planned malaria prevention campaigns had been implemented [17, 28, 54, 57, 59].

### Epidemiologic data from countries

Compared to previous years, fewer papers provided data from African countries on the actual number of malaria cases and deaths in 2020. A small study from Sierra Leone reported a significantly lower number of malaria outpatient visits in one health facility during the March/April 2020 lockdown period compared to the same period in 2019 [29]. In addition, preliminary national data from Uganda point to a reduction of malaria cases diagnosed in health facilities during the first quarter of 2020 compared to the same period in 2019 [61]. Another study from Uganda reported a 54% decrease in visits for malaria treatment of febrile children; visits for antenatal care declined by 26%, restricting the delivery of intermittent preventive malaria treatment in pregnancy (IPTp) [62]. In the Democratic Republic of the Congo (DRC), lower attendance to health facilities for malaria treatment ranged from 20 to 90%, depending on local lockdown measures [63]. In contrast, a study from one rural district in Zimbabwe reported a large increase in malaria cases in 2020 compared to previous years, which was associated with delayed IRS in 2020 [50]. These findings were confirmed by national data from Zimbabwe, which compared the number of malaria cases and deaths in 2020 with those in previous years; in 2020, there was a large excess of reported malaria cases and deaths [27, 64]. Moreover, national data from Zambia showed an increase of malaria cases between August 2019 and June 2020; however, no data from control periods were provided [65].

## Discussion

The COVID-19 pandemic has a massive impact on nearly all countries across the world. While the initial spread of SARS-CoV-2 to Africa has been slow and the COVID-19 burden appears to be much lower than in other continents, the pandemic carries a high potential to negatively affect the control of other diseases, such as malaria [7]. It has already been shown, that the pandemic has resulted in major reductions of incidence of other respiratory diseases due to various effects [66]. Moreover, it has been predicted that the pandemic will result in major disruptions of routine childhood vaccinations, which may cause an increase in vaccine-preventable infectious diseases in SSA [67]. Both malaria and COVID-19 affect disproportionally the low socio-economic classes [28, 32, 68]. It is possible that the COVID-19 pandemic and its indirect effects, including the measures to contain it, may produce collateral damage similar to those seen 6 years ago during the West African Ebola epidemic, i.e., a sharp increase of malaria deaths which finally exceeded the direct Ebola mortality [17, 36, 69]. Thus, understanding how the COVID-19 pandemic affects malaria control measures is of extreme importance for SSA [17, 59].

Accelerated malaria control efforts since the early twenty-first century have significantly reduced the malaria burden in Africa and worldwide [17]. Control strategies include ITN and IRS interventions, early diagnosis and rapid treatment with ACT, and intermittent preventive treatment for infants, children and pregnant women [14]. However, the rate of reduction in malaria morbidity and mortality in SSA has recently stalled, and the initial overall positive trend could be seriously reversed due to the effects of the COVID-19 pandemic as shown in several modelling studies [17, 21–23].

In accordance with the conceptual framework, four major themes likely play an important role for the effects of the COVID-19 pandemic on malaria in SSA: (1) diagnostical and clinical aspects; (2) access to health care services; (3) availability of curative and preventive malaria commodities; and, (4) impact on malaria prevention programmes. While diagnostical and clinical aspects will play an obvious role due to the overlapping symptoms of both diseases [27, 70, 71], therapeutic aspects related to initial misperceptions regarding the efficacy of certain anti-malarials against COVID-19 may have been overemphasized [25, 36]. Co-infection with malaria may complicate COVID-19, while immunomodulation caused by previous malaria exposure may result in less severe COVID-19, as was previously also shown in other respiratory diseases [72–75]. Reduced access to health care services due to direct and indirect effects of the pandemic has a negative impact on access to anti-malarial treatment, thus it would likely have a major effect on the malaria burden in endemic countries [17, 49, 53]. This will be compounded by the clear negative impact of the pandemic on global supply chains for curative and preventive malaria commodities [48, 52]. The consequences of the pandemic for preventive malaria control programmes have been much emphasized by many of the reviewed papers and particularly in modelling papers. However, as an effect of such early warnings, country programmes and funding for malaria have probably adapted rapidly to the pandemic as early as 2020, which may have reduced the modelled impact [28, 59]. International actors such as WHO may have contributed to the prevention of some worst-case scenarios by providing adapted malaria strategies and keeping malaria in their priorities [17, 30].

Until June 2021, only a few reports provided actual epidemiological data on malaria in SSA during the first wave of the pandemic in 2020, thus drawing conclusions on these data might be premature. However, these reports showed that the number of reported malaria cases in Sierra Leone, Uganda and the DRC, that are more highly malaria-endemic countries, was much lower than expected [29, 61–63], while the number of reported malaria cases in Zimbabwe and Zambia, that are countries of low endemicity, was higher than in previous years [27, 29, 64, 65]. It could be speculated that possibly lower access to health care services in combination with impaired malaria surveillance systems may have led to a lower number of reported malaria cases and deaths in these selected highly endemic countries. In the two low-endemic southern SSA countries, disruption of malaria control activities within relatively well-functioning health systems, including surveillance activities, may have resulted in a higher number of reported malaria cases and deaths. More information from other African endemic countries is needed to fully assess such developments [59, 76]. As the COVID-19 pandemic is far from being under control in most low- and middle-income countries, as new and more infectious SARS-CoV-2 variants are emerging, and as SSA countries have limited access to COVID-19 vaccines, dramatic increases in the malaria burden may occur [59, 77, 78]. Although the findings of existing modelling studies are already alarming, the final impact of the pandemic on the malaria burden could be even more devasting [21, 51]. Better education, sensitization and de-stigmatization of both diseases is essential, including emphasis on early care-seeking behaviour, which also needs more community participation [25, 29]. Community health workers should be encouraged to treat all uncomplicated malaria cases in the community and to refer to health facilities only severe cases [51, 79]. As 2020 was a year with many planned malaria prevention campaigns, the negative effects of disrupted programmes would probably last for some years [21, 23]. Fortunately, the international community, including WHO, acted fast to counteract such developments [17]. However, there is the need for more support for SSA countries from the international community and from high-income countries [32]. Malaria, one of Africa’s deadliest diseases, which disproportionally affects the most vulnerable population groups, must be kept under control [16, 35, 59].

## Conclusion

The findings from this review provide evidence for a significant but diverse impact of the COVID-19 pandemic on the malaria burden in SSA. Only results of further studies will enable a full understanding of these developments and its public health consequences. In the meantime, SSA countries need more support from the international community including the urgent delivery of COVID-19 vaccines for high-risk groups.

## Data Availability

The datasets used and/or analysed during the current study are available from the corresponding author on reasonable request.

## Appendix

### Appendix 1: Detailed search strategy

*Concept 1* COVID-19

“COVID-19”[ALL] OR “COVID*”[ALL] OR “SARS-CoV-2”[ALL] OR “coronavirus*”[ALL] OR “2019-nCoV disease”[ALL] OR “betacoronavirus”[ALL] OR “nCoV”[ALL] OR “COVID-19” [Supplementary Concept] OR “severe acute respiratory syndrome coronavirus 2”[nm]

*Concept 2* malaria

“malaria*”[ALL] OR “paludism*”[ALL] OR "Malaria"[Mesh] OR "Malaria/prevention and control"[MAJR]

*Concept 3* sub-Saharan Africa

“africa”[ALL] OR “sub-saharan”[ALL] OR “SSA”[ALL] OR “south of the sahara”[ALL] OR "Africa South of the Sahara”[Mesh]

*Concept 1 AND concept 2 AND Concept 3*

**Table:**

Search number	Query	Sort By	Filters	Search details	Results	Time
**5**	#1 AND #2 AND #3	Most recent	From 2019/12/1–2021/6/9	(("COVID-19"[All Fields] OR "covid*"[All Fields] OR "SARS-CoV-2"[All Fields] OR "coronavirus*"[All Fields] OR "2019-nCoV disease"[All Fields] OR "betacoronavirus"[All Fields] OR "nCoV"[All Fields] OR "COVID-19"[Supplementary Concept] OR "severe acute respiratory syndrome coronavirus 2"[Supplementary Concept]) AND ("malaria*"[All Fields] OR "paludism*"[All Fields] OR "Malaria"[MeSH Terms] OR "malaria/prevention and control"[MeSH Major Topic]) AND ("africa"[All Fields] OR "sub-saharan"[All Fields] OR "SSA"[All Fields] OR "south of the sahara"[All Fields] OR "Africa South of the Sahara"[MeSH Terms])) AND (2019/12/1:2021/6/9[pdat])	117	13:34:11
**4**	#1 AND #2 AND #3	Most recent		("COVID-19"[All Fields] OR "covid*"[All Fields] OR "SARS-CoV-2"[All Fields] OR "coronavirus*"[All Fields] OR "2019-nCoV disease"[All Fields] OR "betacoronavirus"[All Fields] OR "nCoV"[All Fields] OR "COVID-19"[Supplementary Concept] OR "severe acute respiratory syndrome coronavirus 2"[Supplementary Concept]) AND ("malaria*"[All Fields] OR "paludism*"[All Fields] OR "Malaria"[MeSH Terms] OR "malaria/prevention and control"[MeSH Major Topic]) AND ("africa"[All Fields] OR "sub-saharan"[All Fields] OR "SSA"[All Fields] OR "south of the sahara"[All Fields] OR "Africa South of the Sahara"[MeSH Terms])	121	13:33:52
**3**	"africa"[ALL] OR "sub-saharan"[ALL] OR "SSA"[ALL] OR "south of the sahara"[ALL] OR "Africa South of the Sahara"[Mesh]	Most recent		"africa"[All Fields] OR "sub-saharan"[All Fields] OR "SSA"[All Fields] OR "south of the sahara"[All Fields] OR "Africa South of the Sahara"[MeSH Terms]	372,746	13:33:40
**2**	"malaria*"[ALL] OR "paludism*"[ALL] OR "Malaria"[Mesh] OR "Malaria/prevention and control"[MAJR]	Most recent		"malaria*"[All Fields] OR "paludism*"[All Fields] OR "Malaria"[MeSH Terms] OR "malaria/prevention and control"[MeSH Major Topic]	104,127	13:33:32
**1**	"COVID-19"[ALL] OR "COVID*"[ALL] OR "SARS-CoV-2"[ALL] OR "coronavirus*"[ALL] OR "2019-nCoV disease"[ALL] OR "betacoronavirus"[ALL] OR "nCoV"[ALL] OR "COVID-19" [Supplementary Concept] OR "severe acute respiratory syndrome coronavirus 2"[nm]	Most recent		"COVID-19"[All Fields] OR "covid*"[All Fields] OR "SARS-CoV-2"[All Fields] OR "coronavirus*"[All Fields] OR "2019-nCoV disease"[All Fields] OR "betacoronavirus"[All Fields] OR "nCoV"[All Fields] OR "COVID-19"[Supplementary Concept] OR "severe acute respiratory syndrome coronavirus 2"[Supplementary Concept]	158,835	13:33:26

### Appendix 2: Data extraction table

Table of included studies.

**Table:**

Authors and year	Title	Study place	Population	Study design	Outcome
Aborode et al. [48]	Fighting COVID-19 at the expense of malaria in Africa: The Consequences and Policy Options	Sub-Saharan Africa	General population	Opinion paper	Supply chain disruptions; financial shortages; problems for HCWs; changed health-seeking behaviour; simplified modelling studies, real outcome could be worse
Aïkpon et al. [54]	Digitalized mass distribution campaign of ITNs in the particular context of Covid-19 pandemic in Benin: Challenges and lessons learned	Benin	General population	Country report	Benin: successful ITN and IRS campaigns, adapted to COVID-19 hygiene measures
Ajayi et al. [28]	Malaria and COVID-19: Commonalities, intersections and implications for sustaining malaria control	Africa	General population	Opinion paper	COVID-19 and malaria are both related to low socio-economic status; health system and diagnostical challenges; changed health seeking; lack of reliable data due to limited reporting
Amimo et al. [16]	What does the COVID-19 pandemic mean for HIV, tuberculosis and malaria control?	Africa	General population	Report	Clinical and socio-economic aspects; changed health-seeking behaviour
Amimo et al. [53]	The potential impact of the COVID-19 pandemic on HIV, tuberculosis and malaria control in Africa: A systematic review of modelling studies and population surveys	Africa	General population	Review	Malaria programme and antenatal clinic disruptions; antenatal care avoidance; increased costs for malaria services
Anjorin et al. [34]	Co-morbidities and the COVID-19 pandemic dynamics in Africa	Sub-Saharan Africa	General population	Review	Clinical aspects e.g., overlapping age groups, common symptoms; malaria health service disruptions
Ansumana et al. [25]	Effects of disruption from COVID-19 on anti-malarial strategies	Sub-Saharan Africa	General population	Opinion paper	Health system challenges; COVID-19 myths and misinformation affect malaria (reduced ITN usage, increased anti-malarial uptake)
Baral et al. [56]—pre-print	Competing health risks associated with the COVID-19 pandemic and response: A scoping review	General and Africa	General population	Review	73% disruptions among malaria programmes; 62% decrease of malaria diagnoses; delays in malaria surveillance
Bell and Hansen, [24]—pre-print	Relative burdens of the COVID-19, malaria, tuberculosis and HIV/AIDS epidemics in sub-Saharan Africa	Sub-Saharan Africa	General population	Modelling study	Low direct COVID-19 impacts in SSA but high indirect impacts on other diseases such as malaria
Bell et al. [61]	Predicting the impact of COVID-19 and the potential impact of the Public Health Response on Disease Burden in Uganda	Uganda	General population	Modelling study	Reduction of malaria cases, admissions, deaths in Uganda
Beshir et al. [37]	Emergence of undetectable malaria parasites: A threat under the radar amid the COVID-19 pandemic?	General and Africa specific	General population	Opinion paper	Diagnostic challenges for malaria due to mutated parasites; clinical challenges and treatment problems
Brooke et al. [33]	Implementing malaria control in South Africa, Eswatini and southern Mozambique during the COVID-19 pandemic	South Africa, Eswatini, southern Mozambique	General population	Review	Disruptions of malaria programmes; diagnostic, health system and socio-economic challenges
Buonsenso et al. [80]	Child healthcare and immunizations in sub-Saharan Africa during the COVID-19 pandemic	Sierra Leone	Children under the age of 5 years	Country report	Reduction in malaria diagnoses (25–40%); no increases in malaria deaths
Buonsenso et al. [29]	Management of malaria in children under 5 years old during COVID-19 pandemic in Sierra Leone: A lesson learned?	Sierra Leone	Children under 5 years	Country report	Changes in malaria diagnoses at health facilities in context of the lockdown; community education campaign in Sierra Leone; difficult data collection
Burt et al. [62]—pre-print	Indirect effects of COVID-19 on maternal, neonatal, child, sexual and reproductive health services in Kampala, Uganda	Uganda	Mothers and newborns	Country report	Greatest impacts from delayed health seeking, no public transport, HCWs disruptions; closures of health facilities for outpatients; decreased antenatal care impacts IPTp; visits for malaria in children decreased by 54%
Chanda-Kapata et al. [26]	COVID-19 and malaria: A symptom screening challenge for malaria-endemic countries	Africa	General population	Opinion paper	Health system disruptions; clinical aspects; importance of parallel testing; malaria commodities supply disruptions
Chasaya [65]	An update on malaria trends in Zambia (2019 to 2020); A descriptive study	Zambia	General population	Country report	Increased malaria testing and cases
Coker et al. [81]	Things must not fall apart: The ripple effects of the COVID-19 pandemic on children in sub-Saharan Africa	Sub-Saharan Africa	Children aged 0 to 19 years	Review	Malaria elimination is threatened by COVID-19
Di Gennaro et al. [35]	Malaria and COVID-19: Common and different findings	General and SSA-specific	General population	Opinion paper	Changed health-seeking behaviour disproportionately affects children; health system challenges; clinical aspects, co-infections
Diongue and Diallo [82]	COVID-19 during malaria transmission season in Africa and why we should be prepared: An example from Senegal	Senegal	General population	Opinion paper	Changed health-seeking behaviour; clinical aspects; COVID-19 and malaria management challenges
Elliot Mbunge et al. [50]—pre-print	Impact of COVID-19 on malaria elimination: Juxtaposing indoor residual spraying and mobile phones in Buhera Rural District, Zimbabwe	Zimbabwe	General population	Country report	IRS delays in 2020; increase in malaria cases (2981 in 2020, 1376 in 2019); disruptions of health services; anti-malarial resistance problems; challenges for HCWs
Emmanuel Awucha et al. [52]	Impact of the COVID-19 pandemic on consumers’ access to essential medicines in Nigeria	Nigeria	General population	Country report	Increase in alternative medicines uptake (10%) and prizes for anti-malarials; supply chain disruptions; 74% reported less income during the pandemic; LMICs strongly depend on importations
Gavi et al. [64]	Malaria incidence and mortality in Zimbabwe during the COVID-19 pandemic: Analysis of routine surveillance data	Zimbabwe	General population	Country report	16% more malaria cases and 28% more malaria deaths than expected in 2020, probably following several malaria outbreaks
Guerra et al. [57]	Malaria vector control in sub-Saharan Africa in the time of COVID-19: No room for complacency	Sub-Saharan Africa	General population	Opinion paper	Clinical and diagnostical aspects; impacts on vector control measures; malaria campaign delays
Hategeka et al. [63]—pre-print	Impact of the COVID-19 pandemic and response on the utilization of health services during the first wave in Kinshasa, the DRC	Democratic Republic of the Congo	General population	Country report	20–90% reductions in health facility visits for malaria, depending whether the areas had lockdown or not
Hussein et al. [36]	Malaria and COVID-19: Unmasking their ties	General and Africa specific	General population	Review	Hypothesis of causal link between antimalarials usage and low COVID-19 incidence; clinical aspects; challenges for malaria programmes
Inzaule et al. [83]	Genomic-informed pathogen surveillance in Africa: Opportunities and challenges	Africa	General population	Opinion paper	Benefits for malaria from genomics-based surveillance strategy for COVID-19
Kangbai et al. [47]	Re-reading ACT, BCG, and low COVID-19 in Africa	Africa	General population	Opinion paper	Hypothesis: low COVID-19 incidence due to anti-malarial usage and malaria antibodies
Kusotera and Nhengu, [27]	Coronavirus-19 and malaria: The great mimics	Zimbabwe	General population	Country report	Concerns of false positive SARS-CoV-2 Ag-RDTs in malaria infected persons; increase in malaria cases in 2020 (44.7%); overlapping clinical aspects; health system challenges
Makanjuola et al. [84]	COVID-19 and malaria in sub-Saharan Africa: Holistic diagnostic approaches may promote effective clinical case management	Sub-Saharan Africa	General population	Review	Importance of parallel testing for SARS-CoV-2 and malaria; clinical and health system difficulties
Menelas and Sabin [71]—pre-print	Malaria or COVID-19? A case report highlighting a diagnostic challenge in Africa	Rwanda	40 years old woman	Case report	Difficult diagnosis of malaria-COVID-19 co-infection
Mvumbi [85]	Mass intake of hydroxychloroquine or chloroquine in the present context of the Covid-19 outbreak: Possible consequences in endemic malaria settings	General and Africa	General population	Opinion paper	Antimalarials uptake for COVID-19 affect malaria and resistance development
Newby et al. [59]	Global health security requires endemic disease eradication	General and SSA	General population	Opinion paper	Over 90% of malaria campaigns undertaken in 2020; increase of health inequities; benefits of malaria eradication for COVID-19
Nghochuzie et al. [49]	Pausing the fight against malaria to combat the COVID-19 pandemic in Africa: Is the Future of Malaria Bleak?	sub-Saharan Africa	General population	Opinion paper	Anti-malarial resistance; RDTs supply shortages; increases of malaria cases and deaths in Zimbabwe and Cameroon; diagnostic challenges; changed health-seeking and effects on children
US President’s malaria initiative [86]	15 years of fighting malaria and saving lives, Annual Report to Congress, April 2021	Sub-Saharan Africa and Southeast Asia	General population	Report	Seasonal malaria chemoprevention for children in Sahel with minimal delays; community approach for malaria prevention; difficult health care access (nearly 50% of the participants)
Rahi et al. [79]	COVID-19 Mitigation steps provide a blueprint for malaria control and elimination	General and Africa	General population	Opinion paper	COVID-19 control lessons important for malaria management
Rogerson et al.[51]	Identifying and combating the impacts of COVID-19 on malaria	General and Africa specific	General population	Opinion paper	Treatment disruptions; socio-economic aspects; challenges for HCWs; malaria product disruptions; health system challenges, malaria surveillance problems
Rosenthal et al. [87]	COVID-19: Shining the light on Africa	Sub-Saharan Africa	General population	Opinion paper	Financial aspects; anti-malarial shortages
Sherrard-Smith et al. [23]	The potential public health consequences of COVID-19 on malaria in Africa	Sub-Saharan Africa	General population	Modelling study	Malaria deaths in 2020 could double; impact of ITN and anti-malarial disruptions; benefits of seasonal malaria chemoprevention, mass drug administration, presumptive malaria treatment
Shi et al. [55]	Accessing the syndemic of COVID-19 and malaria intervention in Africa	Sub-Saharan Africa (Ethiopia, Nigeria, Tanzania, Zambia)	General population	Modelling study	Greatest impact on malaria health services if COVID-19 waves and main malaria season overlap
Steketee et al. [31]	World Malaria Day 2021: Commemorating 15 Years of contribution by the US President’s Malaria Initiative	General and Africa	General population	Opinion paper	Nearly all malaria campaigns undertaken despite the pandemic; disruptions of malaria testing, and treatment; excess malaria deaths could exceed COVID-19 deaths in some regions
The alliance for malaria prevention [58]	2020 Annual Report	General and Africa	General population	Report	74% of planned ITNs distributed
The Global Fund, 2021 [32]	The impact of COVID-19 on HIV, tuberculosis and malaria services and systems for health: A snapshot from 502 health facilities across Africa and Asia	Africa and Asia	General population	Report	Up to 115 million people in extreme poverty; fear of COVID-19 infection in health facilities as main reason for reduced outpatient visits; malaria diagnosis and treatment reduced by 17 and 15%, respectively; anti-malarials stock-outs (21% of all health facilities), lack of PPE (64%), deficits in COVID-19 testing capacities, lack of malaria treatments (40%); about 75% of malaria programmes reported disruptions; large financial resources needed
Velavan et al. [70]	COVID-19 and syndemic challenges in ‘Battling the Big Three’: HIV, TB and malaria	General and Africa	General population	Review	Increased malaria cases in many countries, suspended vector control activities
Wang et al. [69]	Preparedness is essential for malaria-endemic regions during the COVID-19 pandemic	Africa	General population	Opinion paper	Measures to reduce malaria support the COVID-19 response; health system challenges
Weiss et al. [21]	Indirect effects of the COVID-19 pandemic on malaria intervention coverage, morbidity, and mortality in Africa: A geospatial modelling analysis	Africa	General population	Modelling study	Anti-malarial disruptions with greater impact on malaria incidence and deaths than ITNs; great variability between countries
WHO [22]	The potential impact of health service disruptions on the burden of malaria: A modelling analysis for countries in sub-Saharan Africa	Sub-Saharan Africa	General population	Modelling study	Importance of ITNs; impacts on malaria burden following ITN and anti-malarials shortages; worst case scenario: 769,000 deaths (743,000 in SSA), 70% in children under 5 years
WHO [30]	Tailoring malaria interventions in the COVID-19 response	General and Africa	General population	Policy guideline	Mass drug administration or presumptive treatment of malaria; adaptation of malaria interventions
WHO [17]	World malaria report 2020: 20 years of global progress and challenges, Chapter 10	General and SSA specific	General population	Report and modelling study	Disruptions of malaria health services; delays of malaria programmes; changed health-seeking; adaptation of malaria programmes and guidelines; malaria product shortages; 100,000 additional deaths if 50% anti-malarials disruptions
Zawawi et al. [68]	The impact of COVID-19 pandemic on malaria elimination	General and Africa	General population	Review	COVID-19 challenges Africa’s weak health system; increase in malaria cases; indirect social effects, malaria outbreak in Zimbabwe during the lockdown; clinical challenges

## Abbreviations

ACT: Artemisinin-based combination therapy
COVID-19: Coronavirus disease 2019
CQ: Chloroquine
DRC: Democratic Republic of the Congo
HCW: Health care worker
IPTp: Intermittent preventive treatment in pregnancy
IRS: Indoor residual spraying of insecticides
ITN: Insecticide-treated mosquito net
LMIC: Low- and middle-income country
PICo: Problem, interest, context
PPE: Personal protective equipment
SARS-CoV-2: Severe acute respiratory coronavirus type 2
SSA: Sub-Saharan Africa
WHO: World Health Organization
